# Factors Associated with the Risk of Developing Coronary Artery Disease in Medicated Patients with Major Depressive Disorder

**DOI:** 10.3390/ijerph15102073

**Published:** 2018-09-21

**Authors:** Roger C. M. Ho, Anna C. Chua, Bach X. Tran, Carol C. Choo, Syeda Fabeha Husain, Giang T. Vu, Roger S. McIntyre, Cyrus S. H. Ho

**Affiliations:** 1 Department of Psychological Medicine, Yong Loo Lin School of Medicine, National University of Singapore, Singapore 119228, Singapore; annacyz@gmail.com (A.C.C.); e0157451@u.nus.edu (S.F.H.); 2 Biomedical Global Institute of Healthcare Research & Technology (BIGHEART), National University of Singapore, Singapore 119228, Singapore; 3 Center of Excellence in Behavioral Medicine, Nguyen Tat Thanh University, Ho Chi Minh City 70000, Vietnam; 4 Institute for Preventive Medicine and Public Health, Hanoi Medical University, Hanoi 100000, Vietnam; bach.ipmph@gmail.com; 5 Johns Hopkins Bloomberg School of Public Health, Johns Hopkins University, Baltimore, MD 21205, USA; 6 Vietnam Young Physicians’ Association, Hanoi 100000, Vietnam; 7 College of Healthcare Sciences, James Cook University, Singapore 387380, Singapore; carol.choo@jcu.edu.au; 8 Institute for Global Health Innovations, Duy Tan University, Hanoi 73000, Vietnam; gigi.vugiang@gmail.com; 9 Institute of Medical Science, University of Toronto, Toronto, ON M5S 1A8, Canada; Roger.McIntyre@uhn.ca; 10 Mood Disorders Psychopharmacology Unit, University Health Network, Toronto, ON M5G 2C4, Canada; 11 Department of Psychiatry, University of Toronto, Toronto, ON M5T 1R8, Canada; 12 Department of Toxicology and Pharmacology, University of Toronto, Toronto, ON M5S 1A8, Canada; 13 Department of Psychological Medicine, National University Health System, Singapore 119228, Singapore; su_hui_ho@nuhs.edu.sg

**Keywords:** antidepressants, cytokines, depression, Framingham risk score, Hamilton depression rating scale, pulse wave velocity, triglycerides

## Abstract

*Background:* The aim of this study was to identify factors associated with high Framingham Risk Score (FRS) in medicated patients with major depressive disorder (MDD). *Methods:* We examined 61 medicated patients with MDD (mean age 37.77 ± 7.67, 90.2% women) and 43 non-depressed controls (mean age 38.26 ± 9.20, 90.7% women). We administered the Hamilton Depression Rating Scale (HAM-D) and measured systolic blood pressure (SBP), diastolic BP (DBP), mean arterial BP (MAP), pulse wave velocity (PWV), intima-media thickness (IMT), interleukin-6 (IL-6) and triglycerides. *Results:* We found that medicated patients with MDD had significantly higher levels of HAM-D score (*p* < 0.01), SBP (*p* = 0.015), MAP (*p* = 0.037), IL−6 level (*p* = 0.007), as compared with controls. Medicated patients who remained moderately to severely depressed showed significantly higher SBP (*p* = 0.049), DBP (*p* = 0.009), MAP (*p* = 0.024), IL−6 level (*p* = 0.019), left PWV (*p* = 0.004) and average PWV (*p* = 0.026) than those with mild depression. Multivariate regression showed that the interaction effect between HAM-D score and triglyceride level (*p* = 0.018) was significantly associated with FRS in medicated patients with MDD. *Conclusions:* This study highlights that the interaction effect of the severity of depression and the triglyceride level, was a modifiable factor positively associated with high FRS.

## 1. Introduction

From a public health perspective, depression is the leading cause of disability worldwide and a major contributor to the overall burden of disease [1]. Globally, the point, one-year and lifetime prevalence of depressive symptoms are 12.9%, 7.2% and 10.8%, respectively [2]. The point prevalence of depressive symptoms in women and men are 18–34% and 10–19%, respectively [3]. Women are more likely to suffer from depression and the predisposing factors include low education, lack of a life partner, diminished family function, job stress, unemployment, high anxiety, increased Body Mass Index (BMI), poor nutritional status and job stress [4,5]. Depression is known to be associated with inflammation [6] and cardiovascular diseases [7]. People with depression have an increase in the risk of coronary artery disease (CAD) by 1.5 times more than those without depression [8]. A previous study found that patients with depression were 2.7 times more likely to die from ischemic heart disease (IHD) over a follow-up period of 8.5 years [9].

There are several pathological mechanisms which explain the increased risk of CAD in patients suffering from depression. Psychological stressors increase hemostatic factors and acute phase proteins, leading to thrombus formation and myocardial infarction [7]. Patients with depression tend to have higher inflammatory activities as evidenced by elevated levels of pro-inflammatory cytokines including interleukin (IL)–6 and tumor necrosis factor alpha (TNF–α) [10], higher activity of sympathetic nervous system (SNS) and sympathetic outflow to the heart [11], disruption in the normal circadian rhythm of cortisol secretion [3], endothelial dysfunction [12] and metabolic risk factors [13]. Depression is associated with obesity [14] and visceral fat is the major site for IL-6 secretion [15], which may explain the relationship between depression, inflammation, metabolic risk factors and cardiovascular diseases. Similarly, the IL-6 level is positively associated with systolic blood pressure (SBP) and diastolic blood pressures (DBP) [16]. The increase in blood pressure (BP) may be a stimulus for inflammation and becomes a risk factor for atherosclerotic diseases [16]. There are animal studies suggesting that depression is associated with leptin resistance [17] and an increase in production of neuropeptide Y [18], which can cause an increase in appetite and hypercholesterolemia. Depression-like behavior in hypercholesterolemic mice is accompanied with alterations in the monoaminergic metabolism, providing further evidence to support the association between hypercholesterolemia and depression [19]. Furthermore, specific symptoms of depression, such as insomnia, are associated with hypertriglyceridemia [20].

Pulse wave velocity (PWV) is a cumulative measure of arterial stiffness and peripheral subclinical arteriosclerosis, reflecting changes in endothelial function [21]. The relationship between PWV and depression is unknown in adults who suffer from major depressive disorder (MDD) and receive antidepressant treatment. There is a paucity of research to identify factors positively associated with Framingham Risk Score (FRS) in patients with MDD treated with antidepressants. Nevertheless, the presence of chronic depressive symptoms was found to be associated with higher FRS scores in both HIV-infected and non-HIV infected women [22].

In the present study, we therefore compared the levels of inflammation markers, lipid-related markers and endothelial function between medicated patients with MDD and non-depressed controls as well as medicated patients who remained non-depressed/mildly depressed and moderately to severely depressed. This study had two hypotheses; First, medicated patients with MDD would have higher levels of pro-inflammatory markers (e.g., IL-6), alternation of lipid−related biomarkers and poorer endothelial function than non−depressed controls. Second, medicated patients who remained moderately to severely depressed would have higher levels of inflammation markers, lipid-related biomarkers and poorer endothelial function than those with no or mild depression. The main purpose of this study is to identify factors associated with high FRS in medicated patients with MDD.

## 2. Materials and Methods

### 2.1. Study Setting and Participants

A cross-sectional study was conducted to recruit medicated patients with MDD from the Depressive Disorder Clinic, National University Hospital, Singapore between 1 January 2010 to 31 December 2013. Patients with MDD were included if all of the following inclusion criteria were met: (i) they were aged between 21- and 50-year old (lower age limit, 21 is the legal age for giving voluntary informed consent in Singapore and upper age limit, 50 was set to reduce medical comorbidity in patients with MDD); (ii) meeting the Diagnostic and Statistical Manual (DSM)–IV criteria for MDD and (iii) receiving monotherapy of an antidepressant. Healthy individuals from the community were recruited. These non-depressed controls were carefully selected, such that their age, gender and menopausal status for women were matched with the patients suffering from MDD. The non-depressed controls underwent the same procedures as the patients. Medicated patients with MDD and non-depressed controls were excluded if one of the following exclusion criteria was met: (i) presence of other psychiatric disorders (e.g., schizophrenia, bipolar disorder, dementia); (ii) pregnancy; (iii) post-menopause; (iv) lack of mental capacity to give consent; (v) presence of acute or chronic inflammatory disorders (e.g., lupus, rheumatoid arthritis); (vi) presence of chronic cardiovascular diseases (e.g., heart failure); (vii) presence of organ failure or cancer; (viii) the use of statins or immunosuppressants; (ix) smoking; (x) chronic alcoholism and (xi) substance abuse.

### 2.2. Measures and Instruments

Severity of depression was assessed using the 21-item Hamilton Depression Rating Scale (HAM-D) [23] which is an interviewer-based assessment. HAM-D assesses the following areas: depressed mood, feelings of guilt, suicidal thoughts, insomnia, difficulty in work and activities, psychomotor retardation, agitation, anxiety and symptoms, gastrointestinal somatic symptoms, general somatic symptoms, hypochondriasis, loss of weight, insight, diurnal variation, depersonalization and derealization, paranoid symptoms and obsessional and compulsive symptoms. A score of 0–5 is generally accepted to be within the normal range, 6–10 indicating mild depression, 11–19 indicating moderate depression and 20 or higher indicating severe depression [24]. HAM-D was validated in Singaporeans who suffered from MDD [25].

Blood pressure (BP) was measured by a sphygmomanometer. Each reading was taken three times on the left arm after the participants had been at rest in the sitting position for at least 5 min. Readings were recorded to the nearest even number. The mean value of the three separate readings of systolic blood pressure (SBP) and diastolic blood pressure (DBP) readings were used as the reported BP for each participant. The mean arterial pressure (MAP), which represents the perfusion pressure required to maintain organ perfusion, was calculated using the standard equation MAP = (2/3) DBP + (1/3) SBP (in mm Hg) [26].

Peripheral artery pressure waveforms were obtained noninvasively from the right and left radial arteries using the SphygmoCor device. PWV was measured from Doppler flow signals obtained in sequence from the right carotid and right femoral arteries and subsequently for the left carotid and left femoral arteries. Each site had a minimum of 10 beats using the R wave of the electrocardiogram (ECG) for synchronization. The distance travelled by the pulse wave over the surface of the body was determined (from the sternal notch to the femoral artery and carotid artery to the sternal notch), and PWV was in turn calculated as the distance to transit time ratio, and expressed as meters/second (m/s) [27].

Endothelial reactivity was measured by flow-mediated dilatation (FMD) at the brachial artery using the Prosound Alpha-10 ultrasound system (Aloka Inc., Tokyo, Japan) [28]. A 10 MHz linear array probe, steadied by a stereotactic clamp which allowed for fine positional adjustment, was used to image the brachial artery and position electronic tracking gates at the media-adventitia interface of opposing arterial walls. eTRACKING implemented in this equipment used radiofrequency signals from the tracked B-mode images to provide measurement of vessel distension in real time to 0.01 mm accuracy [29]. Reactive hyperemia was induced by inflation of a pneumatic cuff placed around the proximal forearm to a pressure of 50 mmHg + systolic blood pressure for 5 min. Rapid inflation and deflation was achieved using the E20 Rapid Cuff Inflator (D. E. Hokanson Inc., Bellevue, WA, USA). Proprietary FMD software provided a continuous graphical display of minute vasodilatation from baseline, cuff occlusion, vasodilation and recovery, and automatically calculated parameters such as vessel diameter at maximum dilatation and % FMD. All FMD measurements were performed after abstention from food and exercise for 12 h, coffee and tea for 24 h and alcohol for 48 h. Female subjects were studied at least 7 days after cessation of their last menstrual period, in order to minimize any effect of progesterone on endothelial reactivity. The endothelial activity was measured by a single trained technologist.

The intima-media thickness (IMT) is the distance between the intima and the media of the arterial wall. The IMT of the common carotid and femoral arteries was determined by B-mode ultrasonography using the Aloka Prosound α–10 vascular ultrasound equipment by a trained technologist in accordance with American Society of Echocardiography guidelines [30]. Carotid plaque was defined as localized wall thickening ≥50% of the surrounding vessel wall or as a focal region with IMT >1.5 mm protruding into the lumen [30].

The Framingham Risk Score (FRS) estimates the 10-year risk of developing CAD for adults aged 20 years and older who do not have heart disease or diabetes. The parameters used in the risk calculation are age, gender total cholesterol, HDL, smoking status and SBP. The FRS is a validated cardiovascular risk assessment tool in Singapore [31].

For quantifying cytokine levels in patients and controls, serum was analyzed with a Milliplex^®^ assay (High Sensitivity Human Cytokine kit; Millipore Corp., St. Charles, MO, USA), which allows for the simultaneous quantification of the following human cytokines: IL-6, IL-17 and TNF–α [10,32]. This assay can perform a variety of bioassays including immunoassays on the surface of fluorescent-coded beads known as microspheres. After an analyte from a test sample was captured by the bead, a biotinylated detection antibody was introduced. The reaction mixture was then incubated with Streptavidin-Phycoerythrin conjugate, the reporter molecule, to complete the reaction on the surface of each microsphere. The microspheres were allowed to pass rapidly through a laser, which excites the internal dyes marking the microsphere set. A second laser excited PE, the fluorescent dye on the reporter molecule. Finally, high-speed digital-signal processors identified each individual microsphere and quantified the result of its bioassay based on fluorescent reporter signals. The intra assay coefficient of variation (CV) was 8.1% for IL-6, 4.5% for IL-17 and 10.5% for TNF–α for a concentration range of 3.2 to 10,000 pg/mL, while the inter assay CV was 11.6% for IL-6, 9.9% for IL-17 and 15.9% for TNF–α for a concentration range of 3.2 to 10,000 pg/mL.

High-sensitivity CRP (hs-CRP) is a serum marker of inflammation and atherosclerosis. The hsCRP was assayed on a Roche Cobas Integra 400 Plus Analyser (Roche Diagnostics, Rotkreuz, Switzerland) by a particle-enhanced immunoturbidimetric method. The within-day assay coefficient of variation (CV) was 0.51–2.25% for a concentration range of 0.9 to 18.0 mg/L, while the between day assay CV was 0.86–3.53% for a concentration range of 0.9 to 18.0 mg/L. This serum biochemistry test was carried out at the National University Hospital Department of Laboratory Medicine Laboratory.

Cholesterol (within-day assay CV 0.53 to 1.45% for a concentration range of 2.60 to 7.25 mmol/L, between day assay CV 1.00–1.33% for a concentration range of 2.60 to 7.25 mmol/L), high density lipoprotein (HDL) (within-day assay CV 0.56 to 0.65% for a concentration range of 0.9 to 2.2 mmol/L, between day assay CV 1.18–3.53% for a concentration range of 0.9 to 2.2 mmol/L), and triglycerides (within-day assay CV 0.00–3.85% for a concentration range of 1.6 to 5.1 mmol/L, between day assay CV 1.24–3.40% for a concentration range of 1.6 to 5.1 mmol/L) were measured using a Siemens Advia 2400 (Siemens). Cholesterol was measured using the enzymatic method, HDL using the elimination/catalase method and triglycerides using GPO, Trinder without serum blank.

### 2.3. Statistical Analysis

Results were expressed as mean ± standard deviation (SD) for normally distributed variables or median and range for data which did not follow normal distribution. Continuous data were analyzed using the Student’s *t*-test or Mann–Whitney U test where appropriate. The correlation between the continuous variables were studied by Pearson’s correlation test and Spearman’s rank correlation test for normally distributed data and skewed data respectively. To explore the relationship between two variables, univariate and multiple linear regression analysis were performed. In order to avoid too many variables in the multivariate linear regression analyses, only variables with *p* < 0.05 in univariate linear regression analysis were entered into the multivariate linear regression analysis. Statistical significance was defined as 2-tailed *p*-value < 0.05. All statistical analyses were performed by the PASW (formerly SPSS) program (Version 18.0 for Window 7, Chicago, IL, USA).

### 2.4. Ethics Approval

The study protocol was reviewed and approved by the Domain Specific Review Board (DSRB) of the National Healthcare Group (DSBR reference: 2006/00464). We obtained the written informed consents from participants. Their data were only used for research and kept confidentially.

## 3. Results

### Clinical Characteristics

Sixty-one medicated patients with MDD based on the DSM-IV criteria were recruited. Of these, fifty-five were women (90.2%) and six were men. Fifty-two patients (81.25%) were prescribed with selective serotonin reuptake inhibitors (SSRI). Four patients (6.56%) were prescribed with Noradrenergic and Specific Serotonergic Antidepressants (NASSA). Three patients (4.9%) were prescribed with Serotonin and Noradrenaline Reuptake Inhibitors (SNRIs) and two patients (3.13%) were prescribed with Tricyclic Antidepressants (TCAs). Forty-three age- and gender-matched non-depressed controls were also recruited. Among them, there were thirty-nine women (90.7%) and four men. Characteristics of the patients with MDD and non-depressed controls are shown in Table 1. Depressed patients had a higher HAM-D score (*p* < 0.01), SBP (*p* = 0.015) and MAP (*p* = 0.037) compared with non-depressed controls. DBP, on the other hand, did not differ between the two groups (*p* > 0.05). IL-6, was significantly increased (*p* = 0.007) in patients as compared with controls while there were no significant differences in the levels of the other pro-inflammatory markers including IL-17, TNF–α and hsCRP between two groups. In addition, medicated patients with MDD had a lower HDL level (*p* = 0.043) than controls whereas the cholesterol, triglycerides and LDL levels were not significantly different between two groups (*p* > 0.05). There were also no significant differences in age, proportion of non-smokers and obese participants, BMI, FMD, PWV, and IMT between the two groups (*p* > 0.05).

Table 2 compares the clinical characteristics in among medicated patients with MDD, according to the severity of depression with a HAM-D score of 10 as a cut-off point. Medicated patients who remained moderately to severely depressed were classified as those with a HAM-D score greater than or equal to 10 while those medicated patients with a score less than 10 were considered to be non-depressed or mildly depressed with antidepressant treatment. The results show that the duration of MDD (*p* = 0.004), blood pressure parameters including SBP (*p* = 0.049), DBP (*p* = 0.009) and MAP (*p* = 0.024), IL-6 level (*p* = 0.019), left PWV (*p* = 0.004), average PWV (*p* = 0.026) and FRS (*p* = 0.002) were significantly higher in medicated patients who remained moderately to severely depressed than those who were non-depressed or mildly depressed. The HAMD score was positively correlated with duration of MDD (*p* = 0.002), DBP (*p* = 0.031), IL-6 (*p* = 0.014) and FRS (*p* = 0.038) in medicated patients with MDD.

Table 3 shows that univariate and multivariate linear regression analyses with FRS as dependent variable in medicated patients with MDD. The univariate regression analysis shows the independent variables that were significantly associated with FRS including the HAM-D score (*p* = 0.047), triglyceride level (*p* = 0.016), HAM-D score X triglyceride level (*p* = 0.025), age (*p* < 0.01),), average PWV (*p* < 0.01) and average femoral artery IMT (*p* = 0.002). IL-6 was not associated with FRS (*p* = 0.28). In multivariate linear regression analysis, the interaction effect of HAM-D score and triglyceride level (*p* = 0.018) as well as age (*p* < 0.01) remained significantly associated with FRS (*p* = 0.047) after controlling for average PWV and femoral artery IMT.

## 4. Discussion

Our study compared the levels of inflammation markers, lipid-related markers and endothelial function between medicated patients with MDD and non-depressed controls as well as medicated patients who remained mildly depressed and moderately to severely depressed. We found that medicated patients with MDD had significantly higher BP (SBP and MAP) and serum IL-6 level than non-depressed controls. Medicated patients with MDD also had a significantly lower level of HDL compared with non-depressed controls. The first hypothesis was not fully supported because there was no difference in endothelial function between medicated patients with MDD and non-depressed controls. In medicated patients with MDD, those remained moderately to severely depressed had a significantly longer duration of MDD, higher SBP, DBP and MAP as well as higher IL-6 levels and PWV than those who were non-depressed or mildly depressed. The second hypothesis was not fully supported because there was no difference in lipid-related markers between medicated patients who remained moderately to severely depressed and non-depressed or mildly depressed. Our study investigated for the possible association between clinical parameters and FRS in medicated patients with MDD. Our results showed that the interaction effect between severity of depression and triglyceride level as well as age, remained positively associated with FRS, even after controlling for average PWV and femoral artery IMT.

To our knowledge, this is the second study to examine the relationship between MDD and risk for CAD in Asians, after the initial study was conducted in Korea [33]. Jang et al. [33] found that Korean women with depression showed significantly higher rates of developing intermediate or high risk for CAD when compared with non-depressed women after adjustment for age, SBP, HDL and smoking. In contrast, depression was not associated with intermediate or high risk for CAD in Korean men. In this study, we could not examine the effect of gender because the majority of participants were women (90%). The female predominance is epidemiologically expected because the prevalence of depression among women is higher than men [2]. Among HIV-infected and HIV-uninfected women, the FRS score was higher among women with chronic depressive symptoms as compared with those without depressive symptoms over a period of 9.5 years [22]. O’Neil et al. [34] proposed the addition of depression as a variable to the FRS equation in order to improve the overall accuracy of the model in predicting 10-year risk for CAD in women [34]. Our findings lend support to this proposal.

There are several possible mechanisms by which MDD could increase the risk of CAD. The current study highlights the association between MDD and elevated BP and supports findings from previous study [35]. Previous evidence indicated that stress may impair one’s coping ability and cause MDD [36]. Stress has also been associated with increased BP through the activation of the SNS [37]. An increase in sympathetic discharge leads to vasoconstriction, tachycardia, increased cardiac contractility and secretion of adrenaline [38]. Another probable mechanism by which MDD could lead to CAD is inflammation. Our results show that the IL-6 level is significantly higher in medicated patients who remained moderately to severely depressed. Several studies have shown that pro-inflammatory cytokines are over-expressed in MDD and stress-related disorders [39]. The IL-6, which is secreted during times of stress, is an effective activator of the HPA axis and triggers the release of other pro-inflammatory cytokines [40]. Pro-inflammatory cytokines can cause plaque formation, cardiac irritation and ultimately, CAD [41]. People with MDD may have continuous overexpression of pro-inflammatory cytokines, leading to adverse effects on the cardiovascular system [42].

In this study, medicated patients who remained moderately to severely depressed had significantly higher than average PWV as compared with those who remained non-depressed or mildly depressed. Those remained moderately to severely depressed were poor responders to antidepressant treatment and they had significantly higher PWV. There are three postulated mechanisms leading to increase in PWV in poor responders to antidepressant treatment. The first postulated mechanism would be due to autonomic nervous system (ANS) dysregulation, leading to an increased activation of the sympathetic nervous system (SNS) and a reduced activation of the parasympathetic nervous system. Previous studies suggested that depression is associated with reduced parasympathetic modulation to the heart [43,44]. The second postulated mechanism is neurohumoral activation with increased levels of plasma and urinary catecholamines, especially norepinephrine (NE). An increased concentration of plasma NE is associated with increased level of SNS in patients with MDD [45,46]. The third postulated mechanism would be harmful lifestyle habits including physical inactivity, smoking and unhealthy diet with increased salt intake [47,48,49]. These factors are all associated with increased PWV and arterial stiffness.

The results of this study should be interpreted with caution because of some limitations. First, as our findings were based on cross-sectional data, the temporal order of the association between depression, lipid-related markers, effects of antidepressants and risk of developing CAD cannot be definitively established. Future research should focus on prospective cohort studies as a key of understanding the causality of depression and risk of developing CAD in medicated patients with MDD. A second limitation of the study is that the small sample size of medicated patients with MDD and non-depressed controls reduced the power of the study. Third, most of the study participants were women and this study was not designed to detect the gender differences in the risk of developing CAD among medicated patients with MDD. Fourth, we could not include patients who were drug-naïve or who were experiencing their first episode of MDD without exposure to antidepressants. As MDD is a common psychiatric condition, general practitioners or primary care physicians have started patients on antidepressants before referral to our tertiary specialist center. As a result, we could not recruit non-medicated patients with MDD. In future studies, the inclusion of a sample of antidepressant naive patients with MDD would assist significantly with the interpretation of the findings. Fifth, we did not control for the effect of hemoglobin A1c (HbA1c) levels and sleep parameters, which are associated with the proinflammatory aspects of depression.

Our findings have several clinical implications. First, medicated patients who remained moderately to severely depressed should receive routine screening for CAD. Second, further intervention is required to reduce the risk of hypertension in medicated patients with MDD. Previous research has found that longer exercise time decreased the risk of having depression [50,51] as well as blood pressure, levels of urinary adrenaline and serum cortisol [52]. Third, severity of depression and high triglyceride level may interact synergistically to increase risk of developing CAD in medicated patients with MDD. Psychiatrists should aggressively reduce the interaction effect between severity of depression and triglyceride level by more potent antidepressants and omega-3-fatty acids respectively [53].

## 5. Conclusions

In conclusion, this study highlights that medicated patients with MDD had higher BP and IL-6 than non-depressed controls. Similarly, medicated patients who remained moderately to severely depressed had higher BP, IL-6 and PWV than those who were non-depressed or mildly depressed. Overall, the interaction effect between severity of depression and triglyceride level were modifiable factors positively associated with risk of developing CAD in medicated patients with MDD. From a public health perspective, the findings of our study lend support to include the severity of depression as a variable of the FRS equation in order to improve the overall accuracy for predicting risk of CAD.

## Figures and Tables

**Table 1:** Characteristics of medicated patients with major depressive disorder (MDD) and non-depressed controls.

Variable	Classification of Participants		*p* Value
	Medicated Patients with MDD *n* = 61	Non-Depressed Controls *n* = 43	
Age, years	37.77 ± 7.67	38.26 ± 9.20	0.771
Non-smokers	82.8%	92%	0.331
HAM-D	14 (9, 21)	1 (0, 3)	0 **
BMI, kg/m^2^	24.36 ± 4.98	22.96 ± 4.08	0.131
Obesity (BMI > 30)	9.37%	8%	0.535
SBP, mmHg	114 (106, 126)	108 (100, 116)	0.015 *
DBP, mmHg	72 (67, 79)	68.5 (63, 74)	0.069
MAP, mmHg	84.7 (79.7, 93.3)	82.0 (77.0, 86.3)	0.037 *
Cholesterol, mmol/L	4.93 (4.50, 5.61)	5.26 (4.68, 5.58)	0.329
Triglycerides, mmol/L	1.16 (0.70, 1.60)	0.93 (0.57, 1.37)	0.121
LDL, mmol/L	3.27 (2.72, 3.58)	3.11 (2.75, 3.60)	0.893
HDL, mmol/L	1.37 ± 0.40	1.54 ± 0.32	0.043 *
IL-6, pg/mL	0.20 (0, 0.93)	0 (0, 0.11)	0.007 **
IL-17, pg/mL	0.95 (0, 3.32)	1.91 (0.42, 4.19)	0.154
TNF–α, pg/mL	5.89 (4.71, 8.07)	7.33 (4.20, 10.56)	0.213
hsCRP, mg/L	1.30 (0.50, 4.00)	0.75 (0.40, 2.61)	0.121
sFMD, %	4.42 (2.98, 6.06)	4.40 (2.63, 6.74)	0.768
dFMD, %	4.35 (2.83, 5.82)	4.34 (2.70, 6.59)	0.578
Right PWV, m/s	6.8 (6.1, 7.5)	6.7 (5.9, 7.4)	0.778
Left PWV, m/s	6.8 (5.9, 7.3)	6.5 (5.7, 7.1)	0.357
Average PWV, m/s	6.8 ± 1.1	6.7 ± 1.1	0.491
Right cIMT, mm	0.49 (0.45, 0.59)	0.50 (0.47, 0.60)	0.622
Left cIMT, mm	0.52 (0.47, 0.56)	0.50 (0.45, 0.62)	0.561
Average cIMT, mm	0.52 (0.47, 0.58)	0.50 (0.46, 0.61)	0.995
Right fIMT, mm	0.47 (0.40, 0.56)	0.45 (0.36, 0.47)	0.052
Left fIMT, mm	0.45 (0.39, 0.54)	0.46 (0.40, 0.54)	0.863
Average fIMT, mm	0.47 (0.41, 0.55)	0.45 (0.38, 0.54)	0.330

HAM-D: Hamilton Depression Rating Scale; BMI: Body Mass Index; SBP: Systolic Blood Pressure; DBP: Diastolic Blood Pressure; MAP: Mean Arterial Blood Pressure; LDL: Low Density Lipoprotein; HDL: High Density Lipoprotein; IL-6: Interleukin-6; IL-17: Interleukin-17; TNF–α: Tumor Necrosis Factor-alpha; hsCRP: High-sensitivity C-Reactive Protein; sFMD: Systolic Flow-mediated dilation; dFMD: Diastolic Flow-mediated dilation; PWV: Pulse Wave Velocity; cIMT: Carotid Intima Media Thickness; fIMT: Femoral Intima Media Thickness; Continuous variables are reported as mean ± SD or median (25th, 75th percentile); * Significant at the 0.05 level (2-tailed); ** Significant at the 0.01 level (2-tailed).

**Table 2:** Characteristics of medicated patients who remained as non-depressed/mildly depressed versus those who remained moderately to severely depressed and correlation between clinical factors and Hamilton Depression Rating Scale (HAM-D) Score.

Variables	Severity of Depression of Medicated Patients		*p* Value	Correlation with HAM-D Score	
	HAM-D Score < 10 (Mild Depression) *n* = 17	HAM-D Score > 10 (Moderate to Severe Depression) *n* = 44		Correlation Coefficient	*p* Value
Duration of MDD, months	12.5 (3.5, 30)	60 (12, 96)	0.004 **	0.399	0.002 **
Duration of antidepressant treatment, months	9.5 (2, 16)	9.5 (1.5, 52.5)	0.910	0.008	0.950
Age, years	34.82 ± 8.70	38.91 ± 7.01	0.096	0.092	0.483
BMI, kg/m^2^	22.61 (21.86, 26.59)	24.47 (20.70, 28.31)	0.536	0.091	0.485
SBP, mmHg	110 (106, 116)	115 (107.5, 131)	0.049 *	0.178	0.171
DBP, mmHg	69 (60, 72)	73 (67.5, 81)	0.009 **	0.277	0.031 *
MAP, mmHg	84.67 (73, 85.67)	87.5 (81.33, 98)	0.024 *	0.221	0.086
Cholesterol, mmol/L	4.79 (4.37, 5.37)	5.06 (4.63, 5.39)	0.455	0.009	0.958
Triglycerides, mmol/L	0.89 (0.83, 1.33)	1.19 (0.69, 1.60)	0.957	−0.044	0.789
LDL, mmol/L	3.02 (2.43, 3.56)	3.28 (2.81, 3.50)	0.709	0.044	0.788
HDL, mmol/L	1.31 ± 0.42	1.39 ± 0.40	0.683	−0.119	0.465
IL-6, pg/mL	0 (0, 0.17)	0.33 (0.037, 0.95)	0.019 *	0.312	0.014 *
IL-17, pg/mL	0.95 (0, 4.11)	0.93 (0, 3.12)	0.980	0.024	0.856
TNF–α, pg/mL	5.73 (3.17, 7.25)	6.58 (4.99, 8.50)	0.101	0.192	0.138
hsCRP, mg/L	0.80 (0.50, 2.10)	1.70 (0.50, 4.50)	0.161	0.063	0.628
sFMD, %	3.33 (1.74, 7.02)	4.55 (3.26, 5.49)	0.541	−0.036	0.781
dFMD, %	3.57 (1.52, 6.95)	4.43 (3.02, 5.78)	0.618	−0.063	0.627
Right PWV, m/s	6.58 + 1.06	6.94 + 1.10	0.250	0.086	0.509
Left PWV, m/s	6.10 (5.50, 6.70)	6.95 (6.20, 7.75)	0.004 **	0.202	0.119
Average PWV, m/s	6.34 + 0.89	7.05 + 1.16	0.026 *	0.132	0.312
Right cIMT, mm	0.47 (0.45, 0.60)	0.50 (0.45, 0.58)	0.675	−0.052	0.689
Left cIMT, mm	0.52 (0.48, 0.56)	0.52 (0.47, 0.57)	0.629	0.018	0.888
Average cIMT, mm	0.49 (0.47, 0.58)	0.52 (0.47, 0.57)	0.772	−0.031	0.813
Right fIMT, mm	0.47 (0.39, 0.62)	0.47 (0.40, 0.56)	0.778	0.002	0.986
Left fIMT, mm	0.47 (0.40, 0.55)	0.45 (0.39, 0.53)	0.275	−0.182	0.160
Average fIMT, mm	0.48 (0.42, 0.56)	0.46 (0.40, 0.53)	0.530	−0.094	0.472
FRS	0.65 ± 4.54	5.09 ± 4.96	0.002 **	0.266	0.038 *

MDD: Major Depressive Disorder; HAM-D: Hamilton Depression Rating Scale; BMI: Body Mass Index; SBP: Systolic Blood Pressure; DBP: Diastolic Blood Pressure; MAP: Mean Arterial Blood Pressure; LDL: Low Density Lipoprotein; HDL: High Density Lipoprotein; IL-6: Interleukin-6; IL-17: Interleukin-17; TNF–α: Tumor Necrosis Factor-alpha; hsCRP: High-sensitivity C-Reactive Protein; sFMD: Systolic Flow-mediated dilation; dFMD: Diastolic Flow-mediated dilation; PWV: Pulse Wave Velocity; cIMT: Carotid Intima Media Thickness; fIMT: Femoral Intima Media Thickness; FRS: Framingham Risk Sore; Continuous variables are reported as mean ± SD or median (25th, 75th percentile); * Significant at the 0.05 level (2-tailed); ** Significant at the 0.01 level (2-tailed).

**Table 3:** Univariate and multivariate linear regression analyses using Framingham risk score (FRS) as dependent variable in medicated patients with major depressive disorder (MDD).

Variables	Univariate Linear Regression Analysis	*p*	Multivariate Linear Regression Analysis	*p*
	Slope (SE) ^a^		Slope (SE)	
^a^ HAM-D score X Triglycerides (mmol/L)	0.185 (0.078)	0.025 *	0.126 (0.05)	0.018 *
Age, years	0.541 (0.054)	0 **	0.456 (0.086)	0 **
^b^				
Ave PWV	2.646 (0.494)	0 **	0.645 (0.663)	0.3404
^b^ Ave fIMT	17.875 (5.419)	0.002 **	3.816 (5.319.246)	0.48

HAM-D: Hamilton Depression Rating Scale; PWV: Pulse Wave Velocity; fIMT: Femoral Intima Media Thickness; ^a^ Only variables with *p* < 0.05 are presented here; ^b^ On a logarithmic scale due to skewed distribution; * Significant at the 0.05 level (2-tailed), ** Significant at the 0.01 level (2-tailed).
